# Trends and Challenges in the Detection and Environmental Surveillance of the Hepatitis E Virus

**DOI:** 10.3390/microorganisms13050998

**Published:** 2025-04-26

**Authors:** Mariana Alves Elois, Catielen Paula Pavi, Yasmin Ferreira Souza Hoffmann Jempierre, Giulia Von Tönnemann Pilati, Lucas Zanchetta, Henrique Borges da Silva Grisard, Nerea García, David Rodríguez-Lázaro, Gislaine Fongaro

**Affiliations:** 1 Laboratory of Applied Virology, Department of Microbiology, Immunology and Parasitology, Federal University of Santa Catarina, Florianópolis 88040-900, Brazil; malves@ubu.es (M.A.E.); catielen.p@gmail.com (C.P.P.); yasminfshoffmann@gmail.com (Y.F.S.H.J.); giuliavpilati@gmail.com (G.V.T.P.); lucaszanchetta11@gmail.com (L.Z.); hgrisard@gmail.com (H.B.d.S.G.); gislaine.fongaro@ufsc.br (G.F.); 2 Microbiology Division, Faculty of Sciences, University of Burgos, 09001 Burgos, Spain; 3 Research Centre for Emerging Pathogens and Global Health, University of Burgos, 09001 Burgos, Spain; 4 Department of Animal Health, Complutense University of Madrid, 28040 Madrid, Spain; ngarciab@ucm.es; 5 VISAVET Health Surveillance Centre, Complutense University of Madrid, 28040 Madrid, Spain

**Keywords:** Hepatitis E virus, surveillance, detection, environment, One Health

## Abstract

The Hepatitis E virus (HEV) is responsible for causing Hepatitis E, a zoonotic disease that has emerged as a significant global health concern, accounting for about 20 million infections and 70,000 deaths annually. Although it is often recognized as a disease that is acute in low-income countries, HEV has also been recognized as a zoonotic disease in high-income countries. The zoonotic transmission requires flexible approaches to effectively monitor the virus, vectors, and reservoirs. However, the environmental monitoring of HEV presents additional challenges due to limitations in current detection methods, making it difficult to accurately assess the global prevalence of the virus. These challenges hinder efforts to fully understand the scope of the disease and to implement effective control measures. This review will explore these and other critical concerns, addressing gaps in HEV research and highlighting the need for improved strategies in the monitoring, prevention, and management of Hepatitis E using a One Health approach.

## 1. Introduction

Hepatitis E is a liver inflammation caused by the infection of the Hepatitis E virus (HEV). HEV is one of the most common causes of acute hepatitis in low-income countries. However, recently, it has been recognized as a zoonotic disease prevalent in high-income regions (e.g., Europe), where it is acquired mainly from pigs.

The HEV virion comprises 180 copies of capsid protein arranged in an icosahedral capsid. It has three structural domains: S (shell), M (middle), and P (protruding) [1,2,3]. The most conserved across genotypes is the S domain, which forms the icosahedral shell with a jelly-roll fold, serving as a base upon which the other two domains sit [3,4]. The P domain, involved in receptor binding and immune recognition, is subdivided into P1 (threefold protrusions) and P2 (twofold spikes), which contain β-barrel folds and potential polysaccharide-binding sites [2]. The M domain stabilizes the virion by binding to S and P [5]. The N-terminal region is essential for the native T = 3 structure assembly; its absence results in smaller T = 1 virus-like particles. The C-terminal region, while not affecting morphology, is necessary for RNA encapsidation and virion stability [6].

HEV has a single-stranded RNA with positive polarity, measuring about 27–34 nm in diameter and 7.2 kb in length, which includes short non-coding regions and three open reading frames (ORFs) [7]. It belongs to the *Hepeviridae* family, divided into the *Orthohepevirinae* subfamily, which infects mammals and birds, and the *Parahepevirinae* subfamily, which infects fish [8]. Within the genus *Paslahepevirus*, *Paslahepevirus balayani* includes eight different genotypes, with HEV genotypes 1 to 4 being the most frequently detected.

HEV transmission mainly occurs enterically through fomites, foodborne, zoonotic, and waterborne transmission routes. HEV genotypes 1 (GT1) and 2 (GT2) cause acute hepatitis outbreaks in humans, are predominant in low-income countries and are primarily transmitted through contaminated water sources. Conversely, genotypes 3 (GT3) and genotypes 4 (GT4) are zoonotic and more commonly found in high-income countries, transmitted mainly through raw or undercooked pork or game meat consumption [9,10]. However, in these countries, waterborne transmission can also occur, since GT3 has been detected in untreated and surface waters in European, North and South American, and Asian countries through human and animal waste, including wastewater and agricultural effluent [11,12,13,14].

According to the World Health Organization (WHO), HEV results in about 20 million new infections (3.3 million symptomatic cases) and over 70.000 deaths annually [15].

A systematic review and meta-analysis examining the global prevalence and risk factors of HEV infection found the highest HEV seroprevalence in Africa (21.76%), followed by Asia (15.80%), Europe (9.31%), North America (8.05%), South America (7.28%), and Oceania (5.99%). A global seroprevalence among the general population was estimated at 12.47% [16].

GT1 and GT2 have higher incidence rates in Asia, Africa, the Middle East, and Central America; when detected in Western countries, they are more often associated with travelers who have traveled to HEV-endemic areas. GT3 is present worldwide, while GT4 has become as significant as GT3 in countries such as China, Japan, Taiwan, Hong Kong, and South Korea [17].

The overall mortality rate for Hepatitis E caused by GT1 and GT2 ranges from 0.2 to 4% [9]. However, the most striking feature of GT1 and GT2 is the high mortality rate among pregnant women, which can reach up to 25% [18]. Regarding GT3 and GT4 genotypes, the infection generally presents as asymptomatic. Acute liver failure is rare in patients with GT3 and GT4 infections, regardless of preexisting liver disease, although there are documented reports from European countries [19,20,21,22]. Although preexisting chronic liver disease may predispose individuals to acute-on-chronic liver failure, a study conducted in France and the United Kingdom found a low prevalence of HEV RNA in patients with decompensated chronic liver disease and in patients with severe forms of acute alcoholic hepatitis [23,24]. Therefore, HEV infection may not predispose individuals to more severe liver disease; however, there are case reports documenting instances where liver transplantation was required due to HEV infection [22].

### Virus Replication

**Figure 1:**
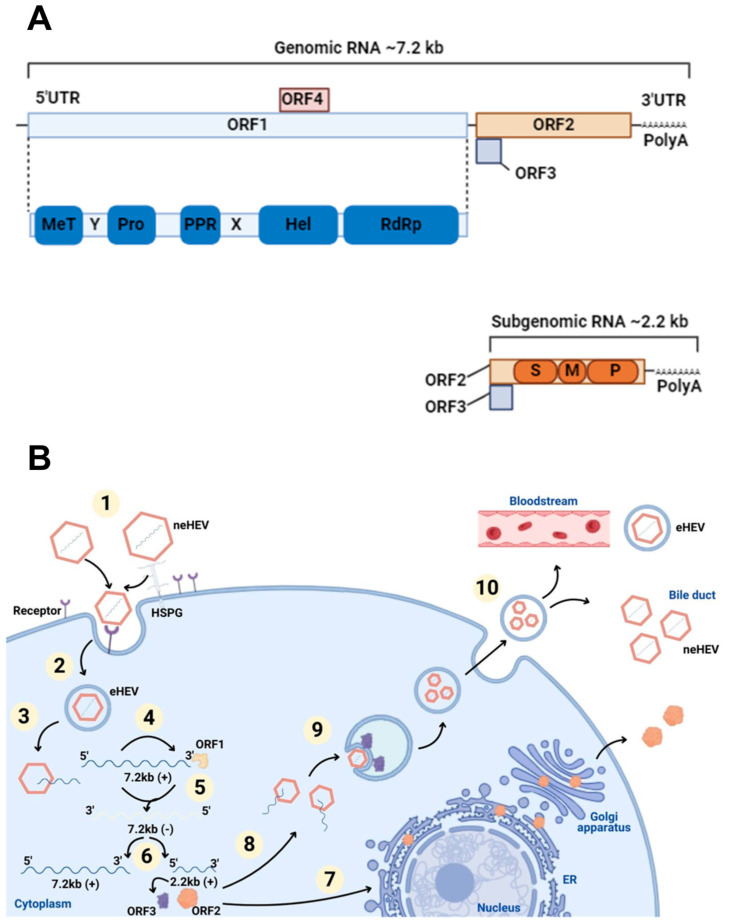
The genome and life cycle of HEV. (**A**) HEV genome. Genomic RNA has a short 5′ untranslated region (UTR), three open reading frames (ORF), a 3′ UTR, and a 3′ polyadenylated (PolyA) tail. ORF1 (blue box) boards the domains: methyltransferase (MeT); Y domain (Y); cysteine protease (PRO); polyproline region (PPR); domain X (X); helicase (Hel); RNA polymerase (RdRp). ORF2 (orange box) and ORF3 (purple box) are translated from subgenomic RNA developed in viral replication. ORF2 contains three domains: S (shell), M (middle), and P (protruding). ORF4 (pink box) is found only in HEV1. (**B**) The life cycle of HEV. (1) Viral attachment: non-enveloped particles of the virus (neHEV) attach to receptors using HSPGs (heparan sulfate proteoglycans) present on the surface of liver cells as an anchoring point; (2) endocytosis: quasi-enveloped HEV (eHEV) particles internalize by clathrin-mediated endocytosis; (3) the release of genomic RNA: positive sense strand is released into the cytoplasm after dissociating from the viral capsid; (4) translation: genomic RNA is used as mRNA for the ORF1 protein production process; (5) RNA replication: a viral replicase contained by ORF1 synthesizes negative-sense intermediate strand RNA that acts as a template for transcription of genomic and subgenomic RNA; (6) translation of ORF2 and ORF3 from subgenomic RNA; (7) non-productive ORF2 pathway: part of the protein produced passes through the endoplasmic reticulum (ER) and is secreted out of the cell; (8) ORF2 productive pathway: the ORF2 capsid protein assembles with the genomic RNA and virions are formed; (9) packaging and assembly: virions are surrounded by a lipid bilayer vesicle and are incorporated into ORF3; (10) virus release: virions are released from cells, by the viroporin activity of ORF3 through the endosomal sorting complexes required for the transport (ESCRT), and can go into the bloodstream as eHEV or into the bile as neHEV. Created in Biorender.

HEV viral RNA is generated as genomic RNA (7.2 kb) and a subgenomic RNA (2.2 kb). The subgenomic RNA initiates near the ORF1 stop codon, enabling the expression of ORF2 and ORF3. ORF1 facilitates viral RNA replication by encoding a polyprotein known as the HEV replicase; ORF2 has a longer form with a signal peptide for secretion (ORF2s) and a shorter form in the capsid (ORF2i). It encodes the capsid protein, which, as previously described, contains the S, M, and P structural domains; ORF3 encodes a phosphoprotein involved in morphogenesis and release of virions [7,10]. In the case of HEV1, it exhibits an additional reading frame, ORF4, which synthesizes a stress-induced protein in the endoplasmic reticulum necessary for the proper functioning of the viral RNA polymerase (Figure 1) [25].

HEV virions exist as non-enveloped (neHEV), secreted in feces, and as qua-si-enveloped (eHEV) virions, found in circulating blood and in the supernatant of in-fected cell cultures. The knowledge regarding the eHEV entry mechanism is poorly understood, but it is known that, upon contact with target cells, neHEV interacts with heparan sulfate proteoglycans. eHEV enters the cell in clathrin-dependent endocytosis via the dynamin-2 and membrane cholesterol pathways [26]. The PI3K pathway and actin were also identified as crucial for virus internalization and intracellular trafficking [27,28].

Once inside the cells, viral RNA is uncoated (a process that remains poorly understood) translated using the host’s translation machinery, while the viral replicase mediates genome replication and release. In short, the viral genomic RNA serves as mRNA for ORF1 polyprotein translation and produces functional enzymes and do-mains. The viral replicase RNA-dependent RNA polymerase (RdRp) synthesizes a complementary negative-sense RNA strand that serves as a template for HEV genome replication and the transcription of subgenomic RNA (sgRNA), which is responsible for the translation of ORF2 and ORF3 proteins [29,30]. The secreted form of the ORF2 protein undergoes post-translational modifications and functions as an immune decoy. In contrast, the capsid-associated form of ORF2 self-assembles into virus-like particles and packages genomic RNA into progeny virions [31]. The ORF3 protein interacts with microtubules and various host cellular proteins to modulate the intracellular environment in favor of HEV replication [32,33]. Moreover, ORF3 binds to the tumor susceptibility gene 101 (TSG101) protein, a component of the ESCRT pathway, facilitating the budding of nascent virions into multivesicular bodies (MVBs) [34]. These MVBs eventually fuse with the plasma membrane, releasing virions into the bloodstream as lipid-enveloped particles (eHEV) or into the bile duct, where bile salts degrade the quasi-envelope [35].

## 2. Sources of Hepatitis E in the Environment and Pathways of Environmental Transmission

### 2.1. Contaminated Water Sources

In low-income countries, the main mode of transmission of HEV is via the fecal-oral route, through contaminated water, which can be attributed to limited access to water, sanitation, hygiene, and health services, as well as inadequate disposal and wastewater treatment [36]. When viral particles are released from feces into the environment, they can directly contaminate drinking, recreational, and surface waters [37]. The last one poses a significant risk to the food production chain since it can indirectly reach shellfish culture and irrigation water, and impact vegetables, fruits, and seafood, to name a few [38]. Furthermore, the vicinity of pig production also represents a contamination risk to surface water, due to runoff waters, percolation events, or agronomic use of pig slurry [39,40]. In addition to inadequate sanitation treatment, the infectious particles in the water are also due to the extremely resistant feature of non-enveloped virions, such as HEV. They are persistent and resistant to several factors, such as drying, UV, and chemical treatments. These factors enhance the virus’s ability to transmit to naïve hosts through the environment and confer distinct advantages of survival and transmission within sensitive populations [41]. 

In high-income countries, HEV-related outbreaks and cases linked to contaminated water are rarely reported. However, some authors highlight a gap in understanding the potential for waterborne transmission in these settings, as studies conducted in France suggest that contaminated water may play a role in the country’s HEV epidemiology [42,43]. 

Table 1 highlights the global burden of HEV outbreaks, demonstrating significant geographical and numerical disparities in its impact. The largest documented outbreak was in China in 1986-1988, affecting approximately 120,000 individuals due to water supply contamination. In comparison, India experienced a large epidemic in 1990–1991 with 79,091 cases, or a reduction of approximately 34% compared to the Chinese outbreak. Similarly, 16,175 cases of HEV occurred in Turkmenistan along with a collapse of the water distribution system in 1985—more than 23 times more cases than in Nepal (692 cases in 1995), which were the result of drinking water contaminated with fecal matter contaminated drinking water. Bangladesh also experienced a major outbreak in 2008–2009, comprising 4751 cases from wastewater contamination of the municipal water supply, while Pakistan had only 133 reported cases in 1987, a difference of nearly 35-fold.

In Africa, Uganda and Somalia had serious outbreaks. Uganda had more than 10,196 cases in 2007, while Somalia had 11,413 cases in 1988–1989, approximately 12% more cases than in Uganda. Both outbreaks resulted from the consumption of untreated river water or public water system breakdowns. These trends show the substantial public health challenge posed by HEV in these regions, likely driven by factors such as inadequate sanitation, and contaminated water supplies. Outbreaks in Mexico, Cuba, or Iraq represented fewer than 300 cases, which may reflect more effective containment measures, improved surveillance, or possible underreporting. Cumulatively, the evidence indicates a strong qualitative correlation between the magnitude of HEV outbreaks and the concurrence of poor water sanitation, dense population centers, and poorly established public health infrastructure—circumstances that, if present, appear to facilitate extensive transmission of the virus through contaminated water supplies. These patterns emphasize the vulnerability of low-income countries to HEV, highlighting the critical need for targeted interventions, including improved water quality, sanitation measures, and access to healthcare.

Apart from the outbreaks, recently, rat HEV, belonging to the *Orthohepevirus C* species, has been reported infecting humans in Hong Kong, Canada, and Spain, including both immunocompromised patients and an immunocompetent individual [89,90,91,92,93]. Additionally, rat HEV has been detected in wastewater, raising concerns about potential environmental exposure [94,95]. While zoonotic transmission from rats to humans has been suggested, the exact sources and routes remain unknown.

### 2.2. Animal Reservoirs and Foodborne Transmission

In 1990, using an experimental model, Balayan reported HEV infection in domestic pigs for the first time [96]. In 1997, the first case of HEV infection in domestic pigs was identified in the USA. This isolate was genetically related to the human HEV strains from the USA and showed cross-reactivity with antibodies against the human HEV capsid protein [97]. Pigs generally do not show clinical symptoms associated with hepatitis, although the presence of hepatitis has already been demonstrated by microscopy [98].

HEV is endemic on domestic swine farms worldwide, and pigs older than three to four months have been found to have antibodies against HEV [99,100]. Since the virus was first identified in domestic swine, many countries have reported finding the virus or antibodies in their pig herds, such as Argentina [101], Brazil [102,103,104], Colombia [105], Czech Republic [106], Denmark [107], France [108], Italy [106], Korea [109], Serbia [110], and Spain [106,111].

The zoonotic potential of genotypes GT3 and GT4 has been well documented, as they have been described in humans as well as in *Sus scrofa domesticus* (domestic pigs) and *Sus scrofa* (wild boars) [98,112] Recently, the range of wild and domestic hosts infected with GT3 and GT4 has increased, and the genotypes circulating in wild ungulates have similarities with human genotypes [98,113].

Among animals in Bulgaria, HEV seroprevalence varies from 32.2% in sheep, 24.4% in goats, 21.1% in dogs, 17.7% in cats, 8.3% in horses to 7.7% in cattle [114]. A study conducted in Irish breeding pigs in 2015 highlighted that 81% of pigs were seropositive, indicating a high HEV infection rate [115]. In the UK, a study reported that 92.8% of pigs were IgG-positive, with 20.5% HEV RNA-positive at the point of slaughter [116].

Over the years, studies conducted in several countries have assessed the presence of HEV genetic material or specific antibodies in wild boars. HEV presence has been confirmed in France [117], Germany [118,119], Italy [120,121,122,123], Netherlands [14], Portugal [112,124], Spain [125,126,127], and Sweden [128].

In 2003, the first identification of HEV genetic material in deer occurred. Along with this identification, the first evidence of foodborne transmission of HEV emerged, associated with an outbreak of acute hepatitis that affected four members of the same family, who consumed raw meat from Japanese deer (*Cervus nippon*) meat [113,129]. After this first report, studies in other countries detected HEV in deer, such as Japan [130,131,132], Spain [125], Netherlands [14], and Uruguay [133].

Several studies have reported evidence of HEV exposure or infection in various animal species, either through the detection of anti-HEV antibodies or HEV RNA, suggesting that these animals may play a potential role in the epidemiology of the virus, such as **cattle** [134,135,136,137,138], **sheep** [135,137,139,140,141], **goat** [137,140,142,143,144,145], **buffalos** [137] and **mooses** [146,147]. In addition to these species, HEV has also been detected in other mammals such as, **rabbits** [148,149,150,151,152,153,154,155], **mongooses** [156,157,158], **ferrets** [159], **camels** [160,161,162], **musks** [163], **rats** [164,165,166,167,168,169,170,171,172,173,174,175], **mice** [176,177], **voles** [176,178], **minks** [179,180], **shrews** [171], **foxes** [181], **bats** [182,183,184], **monkeys** [185,186,187,188], **bisons** [189], **bears** [190], **macaques** [1,188,191,192,193,194,195,196,197,198,199], **cats** [200,201], **chimpanzees** [186,198,202,203,204,205], **leopards** [190], **dogs** [136,200,206,207], **pigs** [97], **donkeys** [208], **brown hares** [153], **langurs** [191], **horses** [209,210], **gerbils** [211], **tamarins** [192], **raccons** [212], **dolphins** [213], **deers** [131,132,210,214,215,216], **muntjacs** [210], **wild boars** [117,119,127,131,189,215,217,218,219,220,221,222], and **yaks** [223]. There are also reports of HEV circulating in **clams** [224], and **mussels** [225,226,227,228]. In addition to **chickens** [229,230], **cranes** [190], **pheasants** [190], **griffons** [231], **buzzards** [232], **ducks** [230], **pigeons** [232], **geeses** [230], **little egrets** [233], **little owls** [232], **thrushes** [232], **sparrows** [234], and **turkeys** [235].

The consumption of raw or undercooked pork products has been identified as a primary risk factor for HEV infection in high-income countries [236]. Products with the highest rate of HEV infection include those made from meat, liver, or viscera when consumed raw or undercooked [98,237]. The virus present in the liver of commercial pigs remains fully infectious and incubating the contaminated meat at a temperature of 56 °C, equivalent to a medium-rare cooking condition, did not completely inactivate the virus [238,239].

Studies from various countries have reported HEV contamination in a variety of meat products. In the food production chain, the slaughter process poses a significant risk of cross-contamination through the use of virus-contaminated tools or handling practices. Di Bartolo [106] detected HEV on the floor, working surfaces, workers hands, and aprons during pig dissection, indicating that the initial production areas (from bleeding to evisceration) are at higher risk for fecal contamination and highlighting potential hazards for workers [106].

Since the first report of foodborne HEV [129], the number of sporadic cases and small outbreaks has increased over the years (Table 2).

The data in Table 2 allow us to recognize patterns in the foodborne spread of HEV, primarily due to the ingestion of raw or uncooked meat products from either domestic or wild animals. The data show foodborne HEV outbreaks that were reported in France and Japan, with at least six separate incidents taking place in Japan alone from 2003 to 2006. The fact that the incidents took place over several years reveals the persistent nature of foodborne HEV threats in the region, typically GT3 or GT4.

In France, figatelli and other pork products were the sources of outbreaks. These were also caused by GT3, once more indicating its zoonotic and foodborne significance in Europe. Notably, an individual French outbreak took place over the years (2007–2009) with at least three cases, showing that certain traditional foods can be ongoing sources of HEV exposure if not properly cooked or processed.

Spain also reported two foodborne cases, one each in 2012 (linked to pork meat) and 2015 (linked to wild boar meat), the latter involving eight individuals, one of the largest foodborne clusters in the table. This outbreak alone accounts for almost 20% of all reported foodborne HEV cases here, indicating the very high risk from consumption of wild game meat.

In South Korea (2009), a single HEV case was associated with the consumption of wild boar bile juice, demonstrating alternative exposures other than through meat, possibly through organ-derived products or traditional medicine. In China (2018), a foodborne outbreak caused by pork meat affected 41 people, which is both the largest outbreak in the table and the only outbreak with more than ten cases. This outbreak is particularly noteworthy because it shows that foodborne HEV outbreaks are not limited to small, localized events, and given the right circumstances—i.e., the wide distribution of contaminated meat—they can take on large proportions. There is one report from the United Arab Emirates (2012), where HEV genotype 7 was identified in a case resulting from the consumption of camel milk. The report is important because it extends the known portfolio of HEV genotypes implicated in foodborne transmission and also because it resurrects questions concerning the role of non-porcine animals in HEV ecology and human infection, especially where raw milk or other animal products are culturally significant.

Trends and conclusions given here are drawn solely from reported events and outbreaks as presented in Table 2. They hence provide the characteristics and outcomes of study samples with documented foodborne HEV transmission events worldwide.

## 3. Current Concentration Methods and Their Limitations and Challenges

The effectiveness of HEV detection in food and environmental matrices is greatly dependent on the viral concentration approach employed, since recovery efficiency directly influences the sensitivity and reliability of downstream analyses. The concentration of HEV in environmental samples faces several overarching challenges, including the wide range of matrices such as water, wastewater, meat, and vegetables, to name a few; beyond the different approaches that must be taken to ensure viral recovery in each one [254,255,256]. The concentration step is essential for virus detection, as viral contamination in food and environmental samples typically occurs at low levels and in highly diluted forms. Furthermore, concentrating viruses from larger sample volumes enhances the sensitivity of detection methods [257,258,259].

The main methods used for HEV concentration include adsorption-elution, ultracentrifugation, filtration, and polyethylene glycol (PEG) precipitation. Membrane-based methods are already consolidated and most used for virus detection in water samples [260,261,262,263]. This method has already been standardized for Norovirus and Hepatitis A, as described in the ISO 15216-1:2017 [264] and it has been tested for HEV detection with adjustments that require a new validation for these specific conditions [37,265]. Briefly, it consists of passing a certain volume of water through a polarized membrane to which the viruses will adsorb via electrostatic interactions. After this, a solution will elute viruses from the membrane. Samples can pass through a secondary concentration process or undergo nucleic acid extraction after elution from the membrane. Depending on the type of wastewater used and how the wastewater is treated, membrane-based methods can be adopted if a clarification step is added to prevent filter clogging [266,267,268,269]. Membrane-based methods often require sample preparation and are highly dependent on the physical-chemical properties of the analyzed sample for optimal viral recovery. Nevertheless, membrane adsorption remains a straightforward and adaptable technique capable of concentrating viruses from substantial volumes of water or wastewater samples, a distinct advantage in detecting HEV.

PEG is a common precipitating agent used in concentration methods for decades in different matrices [270], including multiple HEV concentration protocols. PEG binds to water molecules, forming a protein pellet containing viruses and other proteins [271]. 

In HEV concentration in meat products, PEG also precipitates proteins such as myoglobin and hemoglobin, which can inhibit the PCR reaction [272]. In the same way, in pork livers, the presence of bile salts is a major challenge as it is known to be a PCR inhibitor [273]. To address this, other steps are implemented to clarify the sample and eliminate lipids and proteins that can interfere with further molecular analysis. Clarifying steps may include centrifugation for phase separation, organic solvents to separate the protein phase from the rest of the sample, and emulsifiers for better lipid removal.

Compared to other options, PEG is inexpensive. However, it is a labor-intensive technique that requires time and meticulous handling to execute, potentially affecting the reproducibility of results across laboratories. Related to HEV, PEG methods are shown to be less effective in HEV recovery from meat products compared with ultrafiltration and ultracentrifugation [272,274]. In wastewater, PEG is also shown to have lower recovery rates than ultracentrifugation [275]. However, ultrafiltration and ultracentrifugation are much more expensive techniques requiring costly equipment and resources to execute. 

Ultracentrifugation is used in a variety of matrices for viral recovery [262,276,277]. The technique consists of virus concentration from aqueous solutions and organic homogenates using considerable force to disassociate both the solid and aqueous parts. Ultracentrifugation has already been assessed to recover HEV from environmental samples. This technique effectively clarifies and concentrates viral particles, although it may also co-concentrate impurities, reducing a large volume to a smaller one. Although it can be easily standardized across laboratories, its primary limitation is the high cost of the equipment, making it less accessible in resource-limited settings.

Ultrafiltration is a widely employed method for recovering viruses from environmental samples [278,279,280]. This technique does not require sample preacidification, can simultaneously concentrate multiple pathogens, and achieves high recovery rates [281]. It allows the concentration of large water volumes, up to 1000 liters, but typically necessitates an additional concentration step since the elution process uses 200–500 mL of eluent [282,283,284,285].

The ultrafiltration process is based on size exclusion and includes various approaches, such as dead-end ultrafiltration (DEUF), tangential flow ultrafiltration (TFUF), hollow-fiber ultrafiltration, vortex flow filtration, electronegative filtration, and centrifugal ultrafiltration. DEUF forces water perpendicularly through hollow fiber membranes, trapping particles larger than the pore size, and is particularly suitable for processing large volumes of water [285,286]. TFUF, in contrast, uses a tangential flow to minimize microbial adherence to the membrane, with the retentate concentrating larger particles [287]. Similarly, hollow-fiber ultrafiltration operates in a tangential flow pattern, where viruses exceeding the membrane cutoff are retained and eluted for further analysis [288].

Vortex flow filtration employs Taylor vortices generated by rotating a cylindrical filter, preventing particles from adhering to the membrane surface as water is filtered. Although this automated method shows potential, it is rarely applied for concentrating human pathogenic viruses in environmental water samples [289]. Electronegative filtration, a standard method for concentrating viral particles from wastewater, involves altering the sample’s surface charge to enhance virus binding to electronegative filters [290,291]. Finally, centrifugal ultrafiltration uses centrifugal force to drive liquids through ultrafilters, selectively retaining viral particles while removing smaller contaminants and excess liquid [292]. 

Each variation of ultrafiltration offers unique advantages depending on the sample type and analytical requirements, making it a versatile tool for viral concentration in environmental studies.

### 3.1. Waterborne HEV Studies

The study of Salvador [37] assessed HEV presence in drinking and surface water using three concentration methods. Large water volumes were filtered using NanoCeram® PAC-AG electropositive filters (Nanoceram Filters, Caslte Rock, CO, USA). The filters were eluted with 3% beef extract, and the eluate underwent organic flocculation, followed by sample concentration using Vivaspin® (Sartorius, Göttingen, Germany) concentrators through centrifugation. The results evidenced HEV RNA in 77.8% of surface water and 66.7% of drinking water and infectious HEV in 23.0% of surface water and 27.7% of drinking water [37]. Hennechart-Collette [265] tested a method for detecting Hepatitis A (HAV), HEV, and norovirus in tap and bottled drinking water adapted from the ISO/DIS 16140-4:2018 [293]. In this method, virus particles are concentrated using one filter with a 47 mm positively charged membrane with a pore size of 0.45 μm. The recovery rates for HEV ranged from 27.87% to 53.54% when analyzed from pure RNA extracts based on the water samples. The LOD95 (Limit of Detection) for HEV was determined to be 2.8 genome copies per milliliter of water sample, and the LOQ (Limit of Quantification) was also 2.8 genome copies per milliliter of water sample [265].

Wang [294] described a method to concentrate HEV in raw and still tap water as well as sewage. Firstly, the samples are centrifuged and filtered twice through Nano-Ceram® cartridge filters at an average flow rate of 2.5 L/min, where viruses are electrostatically attached to the filter. Viruses are eluted from the filters and filtered through a Sartobran® Capsule filter (0.65/0.45 μm) (Sartorius, Göttingen, Germany). Finally, the filtrate is ultracentrifuged. In tap water, 10–130 International Units (IUs) of HEV RNA/mL (subtypes GT3a and GT3c/i) were identified [294].

Cuevas-Ferrando [255] evaluated various virus concentration methods for wastewater and drinking water. For influent wastewater, two approaches were used: an ultracentrifugation-based method (UC) and an aluminum hydroxide adsorption–precipitation method (Al). In the UC method, influent water underwent sequential centrifugation and ultracentrifugation. In the Al method, water was treated with aluminum hydroxide, centrifuged, and resuspended in 3% beef extract, with the concentrate recovered by additional centrifugation. For drinking water, the primary concentration utilized a DEUF system, followed by a secondary concentration using PEG precipitation and centrifuge filtration using Amicon^®^ Ultra-15 tubes (Merck KGaA, Darmstadt, Germany). The study evaluated HEV recovery and detection in water samples, finding average recoveries of 15.2%, 19.9%, and 16.9% in influent, effluent, and drinking water, respectively, with detection limits of 10^3^–10^4^ IU/L. A one-year pilot study assessed HEV prevalence in influent and effluent water from four influent and effluent water in waste and two drinking-water treatment plants using three RT-qPCR assays. HEV was detected in 30.65% of influent samples, with an average concentration of 6.3 × 10^3^ IU/L, but not in effluent or drinking-water samples. Despite HEV circulation in the Valencian region, limited assay sensitivity and unknown waterborne infective doses prevented definitive risk assessments of environmental contamination [255].

Wassaf [295] provided HEV detection results by molecular detection on environmental samples, specifically in wastewater samples, using centrifugation and PEG precipitation with HEV (GT3, subtypes a, b, and c) detected in 6.3% of raw sewage samples and 3.2% of riverine samples [295], while Miura’s [296] study used crossflow ultrafiltration and PEG to concentrate effluent samples and recovered concentrations varying from 5.8 to 3.0 RNAc/L, apart from those <LOD (2.2 log RNAc/L) [296].

### 3.2. Foodborne HEV Studies

The study conducted by Son [274] compared different buffer and concentration methods to detect HEV RNA in swine liver samples, including (i) phosphate-buffered saline (PBS, pH 7.4) using ultrafiltration; (ii) threonine buffer using PEG precipitation and ultrafiltration; and (iii) glycine buffer using ultrafiltration and PEG precipitation. Using phosphate-buffered saline ultrafiltration, real-time RT-PCR detected HEV in 6 of the 26 samples. Using threonine buffer with PEG precipitation and ultrafiltration, real-time RT-PCR detected HEV in 1 and 3 of the 26 samples, respectively. When glycine buffer was used combined with ultrafiltration and PEG precipitation, real-time RT-PCR detected HEV in 1 and 3 samples of the 26 samples, respectively. For nested RT-PCR, all samples tested negative regardless of the type of elution buffer or concentration method used [274].

Chatonnat [297] evaluated the presence of HEV RNA in pork liver pâtés and raw pork livers. Pâté was vortexed with internal control virus suspension, TRI Reagent Solution^®^ (Molecular Research Center, Inc., Cincinnati, OH, USA), and centrifuged. Raw pork liver was homogenized with internal control virus suspension, TRI Reagent Solution^®^, RNaseOUT™ (Thermo Fisher Scientific, Carlsbad, CA, USA), and glass beads in a Bead Genie device (Scientific Industries, Bohemia, NY, USA), followed by a centrifugation step. HEV RNA was detected in 29% of the pâtés and 4% of the raw pork livers [297].

The study of Martin-Latil [263] evaluated three virus elution–concentration methods for food samples containing raw pig liver, using three elution buffers: distilled water, PBS (pH 7.4), and TGEB (Tris–HCl, glycine, beef extract, pH 9.5). The methods differ in their handling of viral particles before nucleic acid extraction. In short, Method 1 consisted of sample homogenization in an elution buffer, incubation, and centrifugation, followed by treatment with PEG and 0.3 M NaCl, and centrifugation. Method 2 followed the same steps as Method 1 up to the PEG precipitation and centrifugation. However, the pellet was resuspended in buffer, mixed with chloroform–butanol (1:1), and centrifuged. In Method 3, chloroform–butanol (1:1) was added to the filtrate early to purify viral particles before PEG precipitation and centrifugation. After centrifugation, the aqueous phase containing viruses was subjected to the steps of Method 1 for concentration. The mean recovery rate of HEV from pig liver sausages was 3.94%, and the recovery increased to 18.38% in Figatelli sausages [263].

Randazzo [256] study evaluated methods to concentrate HEV from lettuce and wastewater. For lettuces, Method A was based on the ISO 15216-1:2017 [264], including lettuce elution on TGBE buffer, centrifugation, pH adjusting, PEG and NaCl supplementing, and centrifugation again.

Method B was a modified version of ISO 15216-1:2017 [264], including lettuce elution in TGBE buffer, and PEG supplementing. In Method C, viruses were eluted in lettuce using buffered peptone water (BPW) with Pulsifier equipment. The filtrate was treated with PEG and NaCl, centrifuged, and resuspended in PBS. Sewage samples were concentrated by ultracentrifugation. Irrigation water samples were concentrated by filtration.

The ISO 15216 protocol resulted in average HEV recoveries of 2% in vegetables, while no detection was reported in lettuce or irrigation waters. HEV was detected in sewage only (10/14) [256].

One of the major challenges in both foodborne and waterborne HEV studies is the lack of standardized protocols for viral concentration and detection. In foodborne studies, the wide variety of food matrices—ranging from raw liver to pâtés and cured meats—necessitates the use of diverse methodologies. A similar issue is observed in waterborne studies, where although the impact of the matrix type (e.g., wastewater, river water, drinking water, irrigation water) is generally less pronounced, the same lack of protocol standardization persists. Across both contexts, differences in buffer type, sample size, elution conditions, and RNA extraction kits often result in substantially variable detection rates. Therefore, variations in reported HEV prevalence across studies may reflect as much due to methodological variability as to actual differences in contamination levels, making it essential to harmonize methods for reliable comparison and risk assessment.

## 4. Current Detection Methods and Their Limitations and Challenges

### 4.1. Molecular Techniques

Since pork products are important sources of HEV infection in humans, it is necessary to implement protocols to detect the presence of infectious viruses in pork and pork-derived products [298]. Predominantly, molecular methods for detecting HEV, specifically RT-PCR (Reverse Transcription Polymerase Chain Reaction), have been employed in pork products [299].

Szabo [300] aimed to investigate the distribution of HEV-positive meat products in Germany and carried out different methodologies for homogenizing and extracting sausage samples, followed by identification through real-time RT-PCR. The extraction method using TRI Reagent® yielded the best results. Among the 120 retail samples tested, 21% were HEV-positive, including 20% of raw sausages and 22% of liver sausages. Positive samples were subsequently tested by a nested RT-PCR to get sequences for comparison with other strains. 18 out of 25 were confirmed as HEV-positive, indicating the presence of different viral strain subtypes of GT3. The study showed a high presence of HEV in pork products and optimized extraction protocols, resulting in an efficient methodology for detecting HEV in different types of sausages [300].

It has been found that the RT-qPCR technique achieves greater sensitivity in detecting HEV compared to variations of nested PCR. In this regard, Marziali [301] applied the methodology TaqMan qPCR assay previously described by Jothikumar [302] with internal control (MS2 phage), aiming to study the prevalence of HEV in pig production farms in Argentina, as well as to perform genotyping analysis of positive samples. For this, 135 porcine fecal samples were collected from pigs from 16 different establishments in the central region of Argentina. The genetic material extraction was performed with the NucleoSpin RNA Virus Kit (MACHEREY-NAGEL GmbH & Co. KG, Düren, Germany). HEV detection used the TaqMan qPCR assay targeting a well-conserved sequence within the overlapping HEV ORF2/3 region, and multiplex was used for MS2 detection. The results showed an HEV positivity rate of 8.1% (11/135 samples), with these samples derived from six different locations. The infection rate of confined pigs was 8.7%, compared to 6.3% for outdoor pigs [301].

Son [274] used the Jothikumar protocol [302] and compared it with a nested PCR to determine which methodology had greater sensitivity for detecting HEV. For this, HEV-positive human and porcine fecal samples were used as positive controls, as well as HEV-positive porcine livers. Liver, feces, and porcine bile samples were collected at slaughterhouses. RNA extraction from the samples was performed with the RNeasy Mini Kit (Qiagen, Hilden, Germany). The results showed that 11.1% of bile samples and 12.7% of porcine fecal samples tested positive for HEV using nested PCR. In comparison, 42.2% of bile samples and 15.2% of porcine fecal samples were positive for HEV in real-time RT-PCR. Overall, positive samples varied according to the buffer and molecular method used. The highest detection rate was achieved using PBS as buffer combined with real-time RT-PCR, where 19.2% of liver samples were identified as positive for HEV, and 23.1% of the same ultra-purified samples also tested positive [274].

Porea [303] sought to determine the prevalence of HEV in wild boars from eastern Romania, identifying circulating strains and correlating them with data from previous studies in the region. Fifty samples of liver (45) and spleen (5) from wild boars were collected, and RNA extraction was performed using the RNeasy Mini Kit (Qiagen) with β-mercaptoethanol. To identify HEV-positive samples, the Jothikumar protocol [302] was performed targeting the ORF3 gene. A standard HEV RNA, produced from the transcription of a pCDNA 3.1 ORF2-3 HEV plasmid, was used. Then, nested PCR was performed to amplify ORF2 fragments and allow sequencing analysis of the products. Positive amplicons were sequenced using an internal primer from Nested PCR. The authors found an 18% prevalence of HEV, and all isolates were GT3 [303].

Three of the cited studies employed the Jothikumar protocol [302], albeit with variations in extraction kits and internal controls. The discrepancies in results can be attributed to differences in the matrices sampled and the geographic locations of the studies. Establishing a standardized technique is essential to ensure the comparability of results for specific sample types while minimizing the risk of false-negative and false-positive outcomes. Furthermore, the prevalence of HEV varies between countries, reflecting the circulation of distinct genotypes in different regions.

Zhang [304] explored the prevalence of HEV in Tibetan pig populations in China using nested PCR. From bile samples, RNA extraction was performed with TRIzol™ Reagent (Thermo Fisher Scientific, Carlsbad, USA). cDNA was synthesized for the first round of PCR, along with Forward and Reverse primers targeting sequences of the ORF2 gene and Ex-Taq DNA enzyme. Nested PCR was performed with a set of internal primers and the product of the first PCR. The study revealed 11 positive HEV samples (4.35%), with sequencing revealing GT4 and subgenotype 4b [304].

Among the most modern methodologies based on the detection of genetic material, the RT-RPA (Reverse Transcription Recombinase Polymerase Amplification) technique stands out for its speed in obtaining results, high sensitivity and specificity, and relatively low cost due to the reduced need for equipment, due to the isothermal nature of the reaction [305]

Recent studies, as discussed by Wang [306], involve the development of reverse transcription recombinase polymerase amplification (RT-RPA) techniques targeting the HEV ORF2 gene, integrating fluorescence detection platforms (qRT-RPA) and lateral flow biosensor visible to the naked eye (LFB RT-RPA). The enzyme-based method consists of primer annealing and extension mediated by different enzymes at a constant temperature between 37 and 42 °C. During amplification, amplicons are conjugated to gold particles. Subsequently, the lateral flow test occurs where amplicons move by capillarity and positive samples are visually detected by comparison with controls. Compared to qRT-PCR, the qRT-RPA and LFB RT-RPA assays showed strong agreement, identifying 11 and 12 HEV RNA-positive samples, respectively, out of 14 confirmed positive cases. Their coincidence rates with qRT-PCR were 96.3% for qRT-RPA and 97.5% for LFB RT-RPA. The assays demonstrated resilience to mutations, successfully detecting HEV genotype 4d despite the presence of 4 to 9 nucleotide mismatches in the primers and probes. However, three samples with low HEV RNA levels (Ct values between 34.22 and 37.04) tested negative in the RT-RPA assays but were weakly positive by qRT-PCR. This highlights the slightly lower sensitivity of RT-RPA in detecting low-viral-load samples. The study concluded that RT-RPA assays are effective diagnostic tools for rapid HEV detection, particularly in resource-limited settings, although further validation is required for other HEV genotypes [306].

RPA technology offers several advantages. It operates at low temperatures, such as body temperature, or with simple heating equipment like water baths, making it suitable for use in diverse environments. Its ability to tolerate up to nine mismatches in primers and probes allows for flexibility in detecting viruses with significant genetic variability. Additionally, the reagents are freeze-dried, enabling storage at room temperature for up to six months, which is especially valuable in resource-limited or remote settings. These features make RPA a convenient and rapid alternative for point-of-care diagnostics. However, RPA also presents challenges. The requirement to open reaction tubes for lateral flow biosensor analysis increases the risk of aerosol contamination, particularly in field or laboratory settings with limited containment measures. To mitigate this risk, strict laboratory partitions and frequent use of UV irradiation or DNase treatment are necessary. Handling must also be performed with care, including frequent glove changes and proper tube management. Furthermore, RPA showed slightly lower sensitivity compared to qRT-PCR, as it failed to detect low viral RNA concentrations in some samples. Despite these drawbacks, the speed, ease of use, and low infrastructure requirements make RPA a promising diagnostic tool in various contexts [306].

Using the same technique, Li [307] tested 28 human samples (feces and serum) and 360 rabbit samples (feces and serum). The results showed that when compared to RT-qPCR, RT-RPA-LFS (37 °C for 30 min) was statistically equally sensitive and specific to HEV, with sequencing identifying GT3 and GT4 genotypes [307].

Other isothermal methods useful in HEV diagnosis, which can be applied in the field, include Transcription Mediated Amplification (TMA) and Loop-Mediated Isothermal Amplification (LAMP) [308].

Genetic heterogeneity of HEV is a complicating factor for molecular biology detection assays. Various genotypes could require special adjustments to PCR methodologies or the employment of more generic primers and probes. Genetic diversity can also affect immune responses, complicating the interpretation of serologic tests and may affect the accuracy of prevalence estimates. Furthermore, while being very sensitive and specific, RT-qPCR and nested PCR methods are susceptible to inhibitors in environmental samples such as organic matter or disinfectants used in water treatment. pH, salinity, and detergent concentration can lower the efficacy of RNA extraction and prevent amplification. Therefore, standardizing sample preprocessing and virus-concentration protocols is critical. The lack of standardized practice in laboratories complicates data comparison and the development of a global epidemiological overview.

A key question is the distinction between the detection of mere viral RNA and true infectivity. RT-qPCR is not capable of distinguishing between infectious and non-infectious viral particles and non-viable fragments. When the environmental sample indicates a high viral load, this may not always indicate an immediate risk of infection because most of the particles may be non-viable. Supportive methods such as cell culture or PMA-qPCR can help estimate the infectivity of HEV particles.

### 4.2. Cell-Culture Assays

Cell culture is the gold standard for assessing virus infectivity. However, for HEV, scientists face the absence of efficient cell culture systems and difficulties that require several complex and time-consuming adaptations for effective growth in cell cultures. As a result, the pathogenesis of HEV infection and the basic steps of its life cycle remain understudied [309]. HEV is very difficult to propagate in conventional cell lines and so, there are no standardized methodologies involving cell culture in recognizing HEV infectivity. Although various cell lines and HEV strains have been tested, viral replication is typically slow and results in low virion yields, often leading to non-productive infections [310]. Furthermore, studies on infection in cell cultures have not allowed visualization of viral cytopathic effect, necessitating molecular or immunofluorescence techniques for quantifying infectious HEV, which increases costs [299]. Here, we present some of the cell culture systems developed in the last few years.

Several cell culture systems have been identified for studying human HEV, including HEV isolated from patients, complementary DNA clones, cancer-derived cell lines, primary human hepatocytes, induced pluripotent stem cells, as well as cell culture systems for animal-derived HEV [310]

The HEV viruses used for in vitro and in vivo infection studies can be recovered from patients and are well documented in literature, including the strains Sar-55 (GT1) [53,311], MEX-14 (GT2) [312], 87A (GT1) [44], F23 (GT1) [100], JE03-1760F (GT3) [313], HE-JF5/15F (GT4) [314], TW6196E [315], Kernow-C1 (GT3) [316], and 47832 (GT3) [317]. However, these viruses cannot have their genomes easily altered, presenting a challenge for studying viral protein functions and non-coding regions. In such cases, cDNA clones are utilized.

A pSGI-HEV(I) (GT1) was constructed through subgenomic PCR amplification of hepatitis E outbreak isolates from India. However, in vitro-produced RNA alone failed to infect rhesus monkeys due to the absence of the 5′ cap, which was later identified as essential [318,319,320]. In another study, a pSK-HEV-2-derived was generated via RT-PCR to produce full-length cDNA clones of the HEV isolate Sar-55 (GT1) [319].

A system was also developed to generate swine HEV-derived cDNA clones (pSHEV-1 to -3) by amplifying eight overlapping fragments covering the entire genome, which were subsequently ligated into the pGEM-9zf(-) vector. These clones demonstrated replication competence in Huh-7 cells [321]. Another study synthesized a cDNA clone corresponding to the GT3 strain JE03-1760F (pJE03-1760F/wt) by amplifying three fragments covering the entire genome, which were then ligated into the pUC19ΔAatIISapI vector [322].

For GT4, a cDNA clone (pHEV-4TW) was derived from strain TW6196E by assembling three overlapping genomic fragments. This construct was transfected into a hepatocellular carcinoma cell line, and ORF2 expression confirmed its replication competence. The progeny of this clone also demonstrated the ability to infect HepG2/C3A cells. Notably, both GT3 and GT4 clones are infectious not only in vitro but also in vivo, retaining their ability to infect pigs, their natural hosts [315,323]. 

In addition to these, other studies have also reported the creation of cDNA clones, including a cDNA clone derived from the wild type and adapted GT3 strain Kernow-C1 and a full-length cDNA clone based on the strain G3-HEV83-2-27 [6,324]. Other cDNA clones have been used to construct intergenotypic chimeras to study HEV’s species tropism [323,325].

HEV can infect various immortalized cell lines, including those derived from the colon: Caco-2 [326], Caco-2 subclone C25j [327]; Kidney: BHK-21 (golden hamster) [324], FRhK-4 (rhesus macaque) [325], FRhK-4 (rhesus macaque) in co-culture with infected primary monkey kidney cells [328], LLC-PK1 [316], 2BS [44,329]; Liver: Hepa-RG [330], HepG2/C3A [327,331], Huh-7 [326], Huh-7 subclone S10-3 [332], Huh-7.5 [316], OHH1.Li [316], PLC/PRF/5 [100]; Lung: A549 [329,333], A549/D3 [334]; and Neuron: M03.13, DBRTG, SK-N-MC, DAOY [335], U87 [336], JEG-3, and BeWo [337]. Although tumor-derived cell lines have contributed significantly to advances in HEV research, they present some important limitations. These include their inability to accurately replicate the physiological environment of primary hepatocytes. Additionally, critical cellular factors and pathways that influence authentic virus infection, RNA replication, and progeny virus release may be absent. That said, primary cells, including monkey cells [328,338,339], primary human hepatocytes [340], mouse embryonic fibroblasts [341], stromal cells and decidual and placental explants [342], as well as induced pluripotent stem cells or embryonic stem cells [343] can be utilized to overcome these limitations. 

Several characteristics of HEV contribute to its difficulty in cultivation and replication. As previously mentioned, HEV can naturally exist as neHEV in bile and feces or as eHEV in blood, where it evades neutralizing antibodies. The neHEV is more infectious than eHEV, which explains the difficulty of isolating HEV from blood samples during the acute phase of infection [26]. Furthermore, the different HEV genotypes have varying influences on cell culture systems, which can impact research outcomes and our understanding of the virus. Genotypes 1 and 2 are primarily human-specific and have been more challenging to culture in vitro, showing limited replication in some non-primate cell lines, such as swine kidney cells, albeit with lower efficiency than primate cells [325,344]. Since genotypes 3 and 4 are zoonotic, they have a broader host range and are generally easier to cultivate in cell culture systems. GT3, in particular, has been successfully cultured in various human cell lines and several hepatocarcinoma cell lines, including PLC/PRF/5 and HepG2/C3A, as well as on A549 lung carcinoma cells [310]. 

To enhance in vitro replication, several modifications have been made to optimize the achievement of reliable results, such as supplementing culture media with MgCl2, dimethyl sulfoxide (DMSO), amphotericin B, and FBS, which have increased HEV replication and adaptation in vitro [345,346]. 

Locus [347] tested different cell lines, including human hepatocellular carcinoma, human lung adenocarcinoma, rat neuroblastoma, and porcine liver cells, to determine which was most susceptible to GT3 infection. The supernatant was collected for HEV RNA detection by RT-qPCR seven days post-infection, and cells were conjugated with an anti-HEV capsid antibodies. The A549-D3 cell line showed the highest viral titer in the supernatant, followed by HuH7 and HuH7-S10-3 cell lines, which exhibited the highest percentage of infected cells. Additionally, it was observed that culture in MMEM-10-D medium and the presence of fetal bovine serum (FBS) along with DMSO contributed to viral propagation. In comparative terms, the authors performed molecular techniques aiming at the indirect identification of infectivity by RT-qPCR and achieved better results in terms of assay time, cost, and test sensitivity for methods using PtCl4 and RNAse A to assess capsid integrity, followed by Long-Range RT-qPCR targeting genome integrity [347].

Similarly, Chew [346] investigated the influence of amphotericin B, MgCl2, progesterone, and DMSO on Paslahepevirus species balayani (HEV-A) and Rocahepevirus species ratti (HEV-C1) production in PLC/PRF/5 cells. Improved production was observed when amphotericin B, MgCl2, and DMSO were used from fecal samples with high viral load, with HEV-A genotype 4 remaining infectious for over 18 months when the medium was supplemented with amphotericin B and MgCl2 [346]

In the context of food sample detection, the complex matrix of components further complicates the development of standardization in cell cultures. Stunnenberg [239] implemented a HEV cell culture for the detection of infectious GT3c and GT3e adding dry sausage and liver homogenate inoculation to A549/D3 culture. The method was applied to study the effect of different food processing temperatures on HEV infectivity in pork meat products, demonstrating positive viral detection under different circumstances [239].

In the study by Berto [348], a 3D cell culture methodology was developed using the Rotating Wall Vessel (RVW) tool for detecting infectious HEV. The RVW simulates a more favorable environment for growth and replication in cell cultures, with the authors working with the human hepatocellular carcinoma cell line PLC/PRF/5. Using an HEV-positive pig liver sample, successful HEV replication and high viral titers were observed over 5 months, confirmed by real-time RT-PCR and transmission electron microscopy [348].

The difficulty in isolating HEV via cell culture varies depending on the sample type, genotype, subgenotype, and strain; acute infections tend to hinder isolation due to the low viral titer found. Schilling-Loeffler [349] isolated HEV using an established protocol, with GT3-positive wild boar serum and liver samples in PLC/PRF/5 cell cultures. The first viral passage lasted 3 months, while the second viral passage lasted 6 weeks, with infectivity monitored by RT-qPCR, immunofluorescence, and electron microscopy, as the virus did not exhibit cytopathic effect. The authors successfully isolated two liver samples that originally had high viral titers [349].

In cell culture-based assays, the low viral loads in food samples necessitate maximizing detection potential with minimal processing, emphasizing the need to determine if food homogenates are cytotoxic to HEV-susceptible cells [350]. Developing efficient cell culture systems for HEV is challenging, often requiring months of adaptation for successful in vitro replication, and difficulties in detecting viral infectivity often requiring molecular or fluorescence techniques, thereby prolonging result delivery times [351].

Considering the challenges related to the cell culture system for HEV, animal models provide a valuable alternative for assessing infectivity and transmission risk of environmental HEV strains. Several studies have demonstrated the susceptibility of Mongolian gerbils (*Meriones unguiculatus*) to a broad range of HEV genotypes, including GT1 [352], GT3 [353,354], GT4 [355], GT5, GT7, and rat HEV [356]. The results observed in these studies indicate that the Mongolia gerbil can be a valuable tool for studying the fundamental aspects of HEV. It can provide rapid propagation of wild-type viruses, pathogenesis and mechanisms of HEV replication studies, and potential for preclinical evaluation of antiviral therapies and vaccine candidates.

## 5. Challenges in HEV Surveillance

In Argentina, raw sewage samples (besides clinical samples) were collected between 2016 and 2017 and tested by both real-time and nested PCR to investigate the circulation of hepatitis A and E viruses. HEV was detected in 22.5% of sewage samples, while in 15.9% of clinical samples [357]. Another study in Argentina found HEV RNA (GT3) in 1.6% (3/189) of water samples from the Arias-Arenales River by nested PCR [358]. In Colombia, between 2012 and 2014, samples from drinking water sources and wastewater systems of eight municipalities and two villages was tested for HEV RNA by RT-PCR. HEV genome was present in 23.3% (7/30) of the samples from drinking water sources and in 16.7% (5/30) from sewage, while positive sewage samples were not detected in cell culture [359]. In Brazil, 68 sediment samples, 250 water samples, and 50 samples of pork products (pâté and blood sausage) were tested for HEV genome with a positivity of 36 % for HEV (GT3) for all food tested [360].

Moving to other places in the world, in Italy, a nine-year nationwide environmental surveillance approach (2011–2019) was conducted on 1374 sewage samples. By real-time RT-qPCR, HEV RNA was detected in 74 urban sewage samples (5.4%), while 56 samples were characterized as GT3 strains and 18 as GT1 [361]. In Germany, wastewater from 21 treatment plants and two rivers was collected between 2020–2021 and 605 samples were analyzed for the presence of HEV RNA by real-time RT-qPCR. About 73% of all samples tested positive for HEV RNA and GT3c was the most prevalent variant [362]. In another study, 68/155 (43.9%) of sewage samples collected from 14 wastewater treatment plants in Italy were positive for HEV RNA. In addition, sixty-eight strains exhibited 79.0–91.6% identity to reference sequences of rodent and human origin, clustering into distinct genetic groups with a clear pattern associated with geographic location and specific wastewater treatment plants [94].

Regarding animals, in Spain, serum samples from 425 zoo animals belonging to 109 animal species, including artiodactyls, carnivores, perissodactyls, proboscideans, and rodents were collected from 11 different zoological parks. Anti-HEV antibodies were detected in 36 out of 425. Using a Western blot assay, specific antibodies against GT3 and HEV-C1 antigens were confirmed as ELISA positive. In addition, HEV RNA was not detected in any of the 262 animals tested by RT-PCR [363]. In another study conducted in Italy, serum and fecal specimens from sheep, goats, red deer, roe deer, chamois, and Alpine ibex were collected and screened molecularly and serologically. HEV antibodies were found in sheep (21.6%), goats (11.4%), red deer (2.6%), roe deer (3.1%), and Alpine ibex (6.3%), while HEV RNA was detected in four fecal specimens (3.0%) of sheep [364]. Yoon [365] investigated the prevalence of HEV infection in 283 blood and 114 fecal samples from 397 horses via sandwich enzyme-linked immunosorbent assay and nested RT-PCR. The results revealed positivity for anti-HEV antibodies in 35 serum samples; however, HEV RNA was not detected in any samples [365].

These studies underscore the global circulation of HEV and its presence in diverse environmental and animal reservoirs. Wastewater and environmental surveillance have consistently identified HEV RNA in sewage, rivers, and water sources across Latin America, Europe, and Asia, demonstrating its persistence in various ecosystems. Furthermore, the different detection methods and sampling strategies highlight the need for standardized approaches in surveillance.

Wastewater surveillance serves as a valuable epidemiological tool, offering insights into pathogen circulation. It has been employed to assess community infection trends, evaluate public health interventions, and support timely, evidence-based decisions to reduce the impact of epidemics or outbreaks. Establishing a surveillance system requires collaboration between different agencies, including health agencies, water treatment and management authorities, and environmental agencies for rapid reporting and exchange of information, which can prevent the spread of diseases [366]. In the same way, animal surveillance can also serve as a tool to provide a more comprehensive understanding of HEV circulation and potential sources of zoonotic transmission of HEV.

Regarding clinical cases, in high-income countries, HEV cases are associated mainly with HEV GT3, which is recognized as a zoonotic infection attributed to the consumption of contaminated pork or shellfish products and traced back to contaminated blood products or infected organs [236,367,368]. Based on that, HEV cases have continued to increase, with about 20.000 notified cases from 2005 to 2015 in the European Union/European Economic Area [369]. 

In Europe, there are published studies and high heterogeneity of the surveillance systems across EU/EEA Member States (e.g., distinct levels of awareness, testing, and surveillance efforts). However, there is emerging evidence that HEV has been an under-recognized pathogen in high-income countries over the last decade [370].

In 2017, the WHO Regional Office for Europe published an action plan for dealing with hepatitis in the WHO European Region [371]. This action plan, adapted to the WHO global health sector strategy on viral hepatitis, together with resolution EUR/RC66/R10, was endorsed and signed on 14 September 2016 and aims to eliminate viral hepatitis as a public health threat in the WHO European Region by 2030. One of the main goals was to harmonize surveillance objectives and case definitions by 2018. The goal for 2020 for the Member States was to establish a national hepatitis infection surveillance program to detect outbreaks promptly, assess trends in incidence, inform disease burden estimates, and do effective real-time tracking of viral hepatitis diagnosis, treatment, and care cascade, including vulnerable populations. Based on that, the European Centre for Disease Prevention and Control (ECDC) is supporting EU/EEA countries in implementing the WHO European action plan to enhance or adapt their capacity for HEV surveillance and control [371]. 

The program offers options for the implementation or adjustment of national HEV surveillance and covers criteria derived from the European Association for the Study of the Liver (EASL) for clinical testing, case definitions for acute and chronic HEV infections and reporting schemes, helping countries achieve the goals proposed. These achievements will further improve the knowledge of the epidemiology of HEV infections in the EU/EEA, provide evidence for risk assessment and the implementation of public health measures, and keep noted animal health and food safety authorities. Furthermore, it will support preventive and control measures to reduce the risk of transmission to humans [371].

Implementing surveillance programs for HEV poses several challenges. The first challenge is effective data management since this process is laborious, requiring the transformation from the data collection system to analytical files, appropriate inferences, and interpretation of data. The second major challenge is related to early detection. Complex and diverse matrices, such as blood, organs, and feces, complicate the standardization of techniques due to the need for specific methodologies [372]. Factors such as low viremia in infections and the lack of standardized concentration, extraction, and detection techniques hinder effective HEV control in public health contexts [373]. Therefore, developing new methods and technologies is necessary to harmonize methodology and detection robustness, improving public health emergency responses. A third point is the need for human resources to accomplish analytic data management, statistical analysis, and visualization. It is necessary to train professionals for positions, such as public health data managers and analysts [374].

The widespread detection of HEV in animals, environmental sources, and clinical samples emphasizes the importance of a One Health approach in managing HEV infections.

## 6. One Health Approach

Besides ribavirin, a key drug for chronic HEV infection [375], no other efficient treatment or global vaccination, apart from those implemented in China and Pakistan [376], is well established for Hepatitis E prevention and control. Still, the fact that HEV is vastly disseminated in several hosts besides humans makes managing the disease quite complex. Additionally, possible forestalling actions may vary depending on the geographic region, where in more prevalent areas the infection normally spreads via poor sanitation and hygiene; while in regions with low HEV prevalence, the transmission is mostly zoonotic [377]. 

From an environmental standpoint, water and swine products present themselves as the biggest disseminators of HEV, where viral particles can be transmitted through direct contact with contaminated water or by consuming contaminated or undercooked pork products, as well as other foods exposed to the virus. Even though more studies are required to evaluate the effectiveness of preventive measures globally, managing both water and porcine derivates would go a long way in lessening the transmission rate of HEV infections [36,38,236,377]. 

One of the main proposed prevention actions is personal hygiene. Actions as simple as washing your hands and following biosafety guidelines can help interrupt the transmission chain of HEV. Viral particles can be inactivated by either using chlorine solutions or heating them at 90 °C or higher, so treating water and wastewater, as well as cleaning and thoroughly cooking foods following the above-mentioned criteria also fit within the suggested forestalling strategies for HEV infections. Vaccination would also fit within these actions, but as previously mentioned to there is still no available vaccine for HEV; yet, WHO proposes additional research to achieve a safe and effective vaccine for both affected animals and humans [377,378].

## 7. Conclusions

Different HEV genotypes have been detected in a vast range of mammals, including the main source of HEV transmission to humans from meat products or water-supply sources. Once infected with HEV, humans can develop an acute, but self-limiting hepatitis, yet risk groups such as pregnant people and immunocompromised patients can develop a more hazardous disease possibly leading to death. So, monitoring HEV in humans, animal hosts, and the environment is essential for managing the virus. While hygiene barriers can help prevent dissemination, several limitations remain.

To study HEV in animal samples, firstly, efficient and cost-effective standardized viral concentration methods are needed, since the viral load can be minimal within these samples, as they can be complex matrices with high concentrations of inhibitors. Once concentrated, sensitive, specific, and standardized HEV detection techniques are still required, since the available technologies including molecular biology, serology, and cell-culture-based assays have flaws.

Molecular techniques are limited in their inability to assess infectious viruses, as they only detect genetic material, which complicates studies on HEV survival and inactivation, for example. On the other hand, while cell culture remains the gold standard method for evaluating virus infectivity, it has several limitations. These include specific limitations inherent to each cell-culture system. For example, some cultures are not susceptible to wild-type viruses isolated from patients, as they are often permissive to specific HEV strains. When using tumor-derived cells, an additional challenge lies in accurately replicating the physiological environment of primary hepatocytes.

The main trend of this study is the heterogeneity of surveillance systems, where notification, testing strategy, and report-back systems differ, weakening the ability to measure the actual epidemiological impact of HEV. In addition, surveillance operations are constrained by technical and logistic limitations, primarily the non-harmonization of standards for concentration, extraction, and detection methods, further complicated by the sample matrix heterogeneity—varying from clinical matrices (e.g., organs, blood) to environmental and food matrices. This heterogeneity contributes to variable detection rates and hinders the generation of comparable international datasets. So, although surveillance studies have been conducted globally across different sample types, there is a clear need for the development of new methods and technologies to standardize and harmonize detection approaches, thereby enhancing the robustness of virus monitoring and improving public health emergency responses.

## Figures and Tables

**Table 1:** HEV waterborne outbreaks worldwide.

Country	Detection Method	Number of People Affected	Genotype	Source	Year	Reference
China	Cell culture from the patients’ feces	120,000	GT1	Drinking-water contamination	1986–1988	[44]
Indonesia	-	>2500	-	Supposed river water	1987	[45]
	Sera tested for Anti-HEV IgG	1688	-	Supposed river water	1991	[46]
	Sera tested for Anti-HEV IgG	962	-	-	1998	[47]
Myanmar	Blood samples tested for anti-HEV by ELISA	>111	-	Water supply	1989	[48]
Vietnam	Sera tested for Anti-HEV IgG	261	-	Hau River	1994	[49]
China	Feces analysis on cell culture followed PCR for HEV RNA.	120,000	-	-	1986–1988	[50]
Bangladesh	Blood tested for IgM and IgG antibodies	4751	-	Wastewater contamination of the municipal water system.	2008–2009	[51]
	Sera was tested for IgM antibodies to IgM anti-HEV	200	GT1	Suspected to be the water supply	2010	[52]
Pakistan	-	133	-	Water supply	1987	[53]
	Sera was tested for IgM and IgG anti-HEV	107	-	Water system	1988	[54]
	Anti-HEV IgG and HEV IgM in the tested serum samples	3827	-	Waterborne outbreak	1993–1994	[55]
Nepal	Sera were tested for IgG and IgM to HEV using ELISA tests and PCR were used to detect HEV RNA	692	-	Fecally contaminated drinking water	1995	[56]
Iraq	Blood was tested for HEV IgM antibodies using ELISA	102	-	Water supply	2005	
Turkmenistan	RT-PCR	16,175	GT1	Water distribution system	1985	
India	Stool samples were tested using RT-PCR	79,091	GT1	Water-supply system	1990–1991	[57]
	Anti-HEV IgG and HEV IgM in the tested serum samples	29,000	-	Waterborne outbreak	1955–1956	[58]
	Anti-HEV IgG and HEV IgM in the tested serum samples	2572	-	Waterborne outbreak	1976	
	Anti-HEV IgG and HEV IgM in the tested serum samples	1169	-	Waterborne outbreak	1981	
	Anti-HEV IgG and HEV IgM in the tested serum samples	1072	-	Waterborne outbreak	1982	
	Anti-HEV IgG and HEV IgM in the tested serum samples	118	-	Waterborne outbreak	1984	
	Anti-HEV IgG and HEV IgM in the tested serum samples	3005	-	Waterborne outbreak	1984	
	Anti-HEV IgG and HEV IgM in the tested serum samples	1395	-	Waterborne outbreak	1985	
	Anti-HEV IgG and HEV IgM in the tested serum samples	1015	-	Waterborne outbreak	1986	
	Anti-HEV IgG and HEV IgM in the tested serum samples	2215	-	Waterborne outbreak	1987	
	Anti-HEV IgG and HEV IgM in the tested serum samples	307		Waterborne outbreak	1987	
	Anti-HEV IgG and HEV IgM in the tested serum samples	276	-	Waterborne outbreak	1989	
	Anti-HEV IgG and HEV IgM in the tested serum samples	139	-	Waterborne outbreak	1990	
	Anti-HEV IgG and HEV IgM in the tested serum samples	>3000	-	Waterborne outbreak	1990	
	Anti-HEV IgG and HEV IgM in the tested serum samples	517	-	Waterborne outbreak	1990	
	Anti-HEV IgG and HEV IgM in the tested serum samples	132	-	Waterborne outbreak	1990	
	Anti-HEV IgG and HEV IgM in the tested serum samples	1442	-	Waterborne outbreak	1991	
	Anti-HEV IgG and HEV IgM in the tested serum samples	2427	-	Waterborne outbreak	1993	
	Sera were tested using enzyme immunoassays for IgM antibodies against hepatitis A virus and HEV	1238	-	Untreated drinking water from an unprotected spring	2005	[59]
Egypt	Immunological tests were conducted on stool and blood	56	-	Waterborne outbreak	2007–2008	[60]
Kenya	Synthetic peptide-based enzyme immunoassay (EIA) to detect IgG anti-HEV and Western blot assays to detect IgG and IgM anti-HEV were conducted on serum	1702	-	Primary potable water source	1991	[61]
	ELISA and PCR tests conducted on the patient’s serum	170	-	The authors suggest a possible increase in HEV incidence during the rainy season, with an elevated infection risk in villages relying on river water in the region.	2012	[62]
South Sudan	Sera were tested for IgM and IgG antibody to anti-HEV using Western blot assay	32	-	Outbreak after floods	1988	[63]
	Stool and serum samples were tested for IgG IgM antibody to HEV and for amplification of HEV genome	2621	-	Drinking water	2004	[64]
	The patient’s stool and serum samples were tested for immunoglobulin G and immunoglobulin M antibodies to HEV (serum) and for amplification of the HEV genome (serum and stool)	253	-	-	2004	[65]
	-	989	-	-	2004	[66]
	ELISA anti-HEV IgG and IgM	39 (pregnant women)	-	-	2010–2011	[67]
	Blood specimens were tested for reverse transcription–polymerase chain reaction (rt-PCR) for HEV	5080 cases of acute jaundice syndrome (AJS). 38 out of 62 (61.3%) tested AJS cases were confirmed to be HEV-positive through RT-PCR.	-	Waterborne outbreak	2012–2013	[68]
Central African Republic	Serological test for IgG and IgM anti-HEV antibodies	222	-	Waterborne outbreak	2002	[69]
	Serology IgM	7 (pregnant women)	-	-	2002	[70]
	Serological test for IgG and IgM anti-HEV antibodies	213	-	Waterborne outbreak	2004–2005	[71]
Uganda	Blood was tested for immunoglobulin IgM and IgG against HEV, and HEV RNA by an in-house RT–PCR	>10,196	GT1	-	2007	[72]
	IgM and IgG anti–HEV antibodies	3218	GT1	Person-to-person transmission	2007–2008	[73]
	IgM and IgG anti–HEV antibodies	112	-	Communal hand-rinse and surface-water samples	2007	[74]
	Serums were tested for HEV-specific IgM and IgG and HEV RNA by RT-PCR	987	-	Waterborne outbreak	2009–2012	[75]
Chad	Sera were tested for HEV antigen (anti-HEV Ag) by a fluorescent antibody blocking assay	23	-	Water outbreaks	1983–1984	[76]
	Fecal suspensions were tested by antibody capture of the virus and RT-PCR	12	-	-	1983–1984	[77]
	-	38	-	Presumed waterborne outbreak	1983–1984	[78]
Republic of Djibouti	IgG and IgM-specific antibodies	43	-	Waterborne outbreak	1993	[79]
Algeria	Blood was tested by RT-PCR	23	GT1	-	1986–1987	[80]
Namibia	RT-PCR	9	-	-	1983	[81]
	HEV IgG using the Abbott HEV EIA kit and RT-PCR	>600	GT2	Suspected to be the water supply	1995–1996	[82]
Morocco	Recombinant antigen-based enzyme immunoassay (anti-HEV IgG) and reverse transcription polymerase chain	58	-	Drinking water	1994	[83]
Somalia	anti-HEV IgG	11,413	-	River water	1988–1989	[84]
	Serum samples were tested for IgM and IgG antibodies to HEV	145	-		1988–1989	[85]
Ethiopia	Sera tested against Hepatitis E virus (anti-HEV)	>750	-	Waterborne outbreak	1988–1989	
Northern Cameroon	Serum samples tested for anti-HEV immunoglobulin G (IgG) and M (IgM) with enzyme-linked immunosorbent assay HEV IgG enzyme-linked immunosorbent assay (ELISA) and HEV IgM ELISA 3.0 kits	33	-	Waterborne outbreak	2013	[86]
Mexico		223	-	Waterborne outbreak	1986	[87]
Cuba	IgG and IgM-specific antibodies and RT-PCR	20	GT1	-	1999	[88]
	IgG and IgM-specific antibodies and RT-PCR	26	GT1	-	2005	

**Table 2:** Foodborne cases and outbreaks related to HEV worldwide. * Raw pork liver sausages.

Year	Country	Type of Product	Genotype	Number of Cases	Reference
2003	Japan	Deer meat	3	4	[129]
2003	Japan	Wild boar liver	4	2	[240]
2004	Japan	Wild boar meat	3	5	[241]
2005	Japan	Wild boar meat	3	1	[242]
2006	Japan	Wild boar meat	3	1	[243]
2006	Japan	Pork meat and entrails	4	3	[244]
2006	France	Dried domestic pork meat	3	2	[245]
2007	France	Figatelli *	3	3	[246]
2008				2	
2009				1	
2009	South Korea	Raw bile juice from a wild boar	4	1	[247]
2012	Spain	Pork meat	3	1	[248]
2012	United Arab Emirates	Camel milk	7	1	[249]
2013	France	Figatelli	3	2	[250]
2013	France	Raw pig meat	3	3	[251]
2015	Spain	Wild boar meat	3	8	[252]
2018	China	Pork meat	4	41	[253]

## Data Availability

No new data were created or analyzed in this study. Data sharing is not applicable to this article.

## Abbreviations

The following abbreviations are used in this manuscript:

HEV	Hepatitis E
HAV	Hepatitis A
ORFs	Open reading frames
GT1	Genotype 1
GT2	Genotype 2
GT3	Genotype 3
GT4	Genotype 4
WHO	World Health Organization
PEG	Polyethylene glycol
DEUF	Dead-end ultrafiltration
TFUF	Tangential flow ultrafiltration
IU	International Units
UC	Ultracentrifugation-based method
RT-PCR	Reverse Transcription Polymerase Chain Reaction
RT-RPA	Reverse Transcription Recombinase Polymerase Amplification
qRT-RPA	Reverse transcription recombinase polymerase amplification
LFB RT-RPA	Lateral flow biosensor
TMA	Transcription Mediated Amplification
LAMP	Loop-Mediated Isothermal Amplification
neHEV	Non-enveloped virus
eHEV	Quasi-enveloped virus
RVW	Rotating Wall Vessel
ECDC	European Centre for Disease Prevention and Control
FBS	Fetal bovine serum
DMSO	Dimethyl sulfoxide
